# A Systems Biological View of Life-and-Death Decision with Respect to Endoplasmic Reticulum Stress—The Role of PERK Pathway

**DOI:** 10.3390/ijms18010058

**Published:** 2017-01-05

**Authors:** Margita Márton, Anita Kurucz, Beáta Lizák, Éva Margittai, Gábor Bánhegyi, Orsolya Kapuy

**Affiliations:** 1 Department of Medical Chemistry, Molecular Biology and Pathobiochemistry, Semmelweis University, 1094 Budapest, Hungary; marton.margita@med.semmelweis-univ.hu (M.M.); kurucz.anita@med.semmelweis-univ.hu (A.K.); lizak.beata@med.semmelweis-univ.hu (B.L.); banhegyi@eok.sote.hu (G.B.); 2 Institute of Clinical Experimental Research, Semmelweis University, 1085 Budapest, Hungary; margittai.eva@med.semmelweis-univ.hu

**Keywords:** autophagy, apoptosis, endoplasmic reticulum stress, dynamical behavior

## Abstract

Accumulation of misfolded/unfolded proteins in the endoplasmic reticulum (ER) leads to the activation of three branches (Protein kinase (RNA)-like endoplasmic reticulum kinase [PERK], Inositol requiring protein 1 [IRE-1] and Activating trascription factor 6 [ATF6], respectively) of unfolded protein response (UPR). The primary role of UPR is to try to drive back the system to the former or a new homeostatic state by self-eating dependent autophagy, while excessive level of ER stress results in apoptotic cell death. Our study focuses on the role of PERK- and IRE-1-induced arms of UPR in life-or-death decision. Here we confirm that silencing of PERK extends autophagy-dependent survival, whereas the IRE-1-controlled apoptosis inducer is downregulated during ER stress. We also claim that the proper order of surviving and self-killing mechanisms is controlled by a positive feedback loop between PERK and IRE-1 branches. This regulatory network makes possible a smooth, continuous activation of autophagy with respect to ER stress, while the induction of apoptosis is irreversible and switch-like. Using our knowledge of molecular biological techniques and systems biological tools we give a qualitative description about the dynamical behavior of PERK- and IRE-1-controlled life-or-death decision. Our model claims that the two arms of UPR accomplish an altered upregulation of autophagy and apoptosis inducers during ER stress. Since ER stress is tightly connected to aging and age-related degenerative disorders, studying the signaling pathways of UPR and their role in maintaining ER proteostasis have medical importance.

## 1. Introduction

The endoplasmic reticulum (ER) is a key eukaryotic organelle involved in various metabolic processes (such as gluconeogenesis and lipid synthesis) and it has an important role in maintaining intracellular calcium homeostasis. ER also acts as an essential integrator of external and internal cellular stimuli by keeping the proper balance of secreted and membrane protein level [1,2,3]. Most secreted and plasma membrane proteins are folded and matured in the ER lumen, before being transferred and displayed on the cell surface or released extracellularly. The accumulation of damaged or not properly folded proteins in the ER lumen leads to harmful ER stress [1,2,4]. ER stress might be generated by aging, genetic mutations or environmental factors resulting in various genetic (i.e., diabetes, inflammation) or degenerative disorders (such as Alzheimer’s and Parkinson’s disease) [5,6,7].

Many scientific results have proved that autophagosome formation is immediately accelerated in the presence of ER stress. This observation is also confirmed by increasing autophagic function [8]. Since autophagy has an essential role in promoting cellular-survival during starvation by “self-eating” of parts of the cytoplasm and intracellular organelles, it was claimed that autophagy also has also a crucial protective role after ER stress [9,10,11]. The autophagy-dependent survival is always followed by apoptosis-dependent cell death with respect to severe ER stress revealing a strict order of autophagy and apoptosis over time [8,12]. Although the details of this regulatory system are still unknown, a small model of the underlying control network was suggested by our lab. This model supposes a double-negative feedback loop between the two mechanisms guaranteeing that autophagy and apoptosis cannot be active at the same time, i.e. the sigmoid induction of the survival mechanism is always followed by the switch-like activation of the suicide loop [12,13].

The precise balance between production and consumption of folded proteins is tightly regulated by a complex network of signal transduction pathways, referred to as unfolded protein response (UPR) [4,14]. The primer role of UPR is to avoid cell damage in response to tolerable ER stress, while cell death is induced at excessive level of ER stress. In yeasts, the signaling pathway of UPR has only one well-defined inducer, called IRE-1 (inositol requiring kinase 1) [15]; however, in mammalian cells, the response mechanism seems more complicated and divergent via using three branches of UPR [2]. These transducers activated by ER stress are called IRE-1, PERK (PKR-like ER kinase) and ATF6 (activating transcription factor 6), respectively [2,4,14]. All three components are ER-resident transmembrane proteins, which become active by the same BIP/Grp78 protein with persistent ER stress. While the activation of both IRE-1 and ATF6 promotes transcription of UPR target genes (such as chaperones), the PERK-controlled pathway leads to the general inhibition of protein translation [2,4,14].

IRE-1 is activated by homodimerization and trans-autophosphorylation during ER stress [14]. This process induces its RNase activity which is able to initiate the unconventional splicing of a transcription factor, known as the X-box binding protein 1 (XBP1s) [16,17]. The active XBP1s promotes cell survival by regulating the transcription of various genes involved in protein folding, ER-associated degradation (ERAD), protein quality control, and phospholipid synthesis [17]. XBP1s is able to induce autophagy/ERAD by activating AT-1, an ER membrane transporter, which is essential for the maintenance of the level of acetyl-CoA in the ER lumen [18]. It has been also shown that transient overexpression of XBP1s induces autophagy and promotes proliferation in bone marrow-derived macrophages, while excessive level of XBP1s leads to apoptotic cell death [19]. These results suggested that splicing of XBP1 might be connected to autophagy-apoptosis crosstalk during ER stress. The IRE-1-dependent transcriptional upregulation of many autophagy receptor genes (i.e., p62, LC3) was observed during ER stress [20]. Activation of IRE-1 also promotes the cJUN N-terminal kinase (JNK) signaling pathway required for apoptotic cell death [14,17].

PERK, activated by homodimerization and trans-autophoshporylation, is able to phosphorylate the translation initiation factor eiF2α (Eukaryotic translation Initiation Factor 2 α) [14]. The global protein synthesis is reduced by eiF2α-dependent phosphorylation, therefore decreasing the flux of protein entering the ER [21]. Interestingly, a transcription activator, ATF4 (activating transcription factor 4), is enhanced by the phosphorylation of eiF2α [22]. Two downstream targets of ATF4 were discovered: Gadd34 (growth arrest and DNA damage-inducible 34) [23] and CHOP (transcription factor C/EBP homologues protein) [24], respectively. CHOP-deleted cells are much less sensitive to ER stress compared to wild-type strain [25], while overexpression of CHOP results in cell cycle arrest and/or apoptosis [26]. Some results were recently suggested that beside apoptosis induction, CHOP is also involved in the activation of a number of autophagy genes essential for survival during starvation [27]. Therefore, a dual role is assumed for CHOP to determine the life-and-death decision of cells in response to amino acid starvation. Gadd34 is a regulatory subunit of PP1 phosphatase [28] and its regulation seems to be even more complex. Gadd34 is not only activated by ATF4, but its transcription is also promoted in a CHOP-dependent manner [29,30]. Although PERK stays active during ER stress, the level of eiF2α-P decreases due to Gadd34-dependent dephosphorylation [23,30]. Some experimental data assume that the dephosphorylation of eiF2α-P has a crucial protective feature by enhancing adaptation to ER stress [30].

PERK^−/−^ cells are hypersensitive to the lethal effect of ER stress, thereby suggesting their essential role in the stress-response mechanism [31]. Inhibition of PERK in HEPG2 cells resulted in a drastic decrease of viable cells via apoptotic cell death [32]. However, Cao et al. have revealed that silencing of PERK with shRNA decreased the apoptotic index under saturated fatty acid-induced cellular stress [33]. Zhang et al. have also shown that siPERK increases cell viability when ER stress was generated by addition of silver nanoparticles [34]. Conversely, some data indicate that PERK silencing does not cause more cell death in regards to ER stress [35]. The effect of PERK depletion seems to be controversial with the use of various cell lines and cellular stressors, thus suggesting that further investigation of the exact role of the PERK pathway in ER stress is required.

Traditionally, IRE-1 was considered a positive regulator of cell survival, since artificially sustained IRE-1 activity enhances cell viability [36]. Upton et al. assumed that only IRE-1 is required and sufficient to trigger apoptotic cell death during ER stress while PERK is dispensable in apoptosis induction [37]. However, many experimental data revealed that both pathways have an important role in controlling autophagy-apoptosis crosstalk with respect to ER stress. Both IRE-1 and PERK pathways are essential for the transcriptional upregulation of various autophagy genes, such as *p62*, *NBR1*, *NIX* [20]. Inhibition of either IRE-1 or PERK abrogates the expression of these autophagy genes with respect to ER stress [20]. Beside the regulation of apoptotic genes (i.e., *BIM* and *Bcl-2*) by IRE-1-induced JNK [14,38], the PERK-activated CHOP also controls gene transcription involved in apoptosis, such as *BIM* and *BH3-only proteins* [39,40]. Novel scientific results have revealed that IRE-1 and PERK pathways are not independent from each other, rather a close regulatory connection is observed between them with persistent ER stress. It was also shown that IRE-1 inhibition reduced the upregulation of CHOP suggesting that the IRE-1 pathway has a positive effect on the apoptosis inducer of the PERK pathway during ER stress [20]. Whether the PERK pathway can influence IRE-1 has not been studied yet.

In this study we are focusing on the crosstalk between IRE-1 and PERK branches with respect to ER stress. Since the control network of life-and-death decision is tightly regulated, we approach the problems from a systems biological aspect, using the techniques of both molecular and theoretical biology. We claim that silencing of PERK during ER stress increases cell viability. We also show that the apoptotic cell death induced by IRE-1-activated JNK is abrogated in the siPERK cell line assuming a positive feedback loop between the PERK and IRE-1 arms of UPR. Our mathematical model reveals the essential role of this positive feedback loop between IRE-1 and PERK in determining the dynamical characteristic of life-and-death decision with persistent ER stress.

## 2. Results

### 2.1. Silencing of PERK Increases Cell Viability with Respect to Endoplasmic Reticulum (ER) Stress

**Figure 1:**
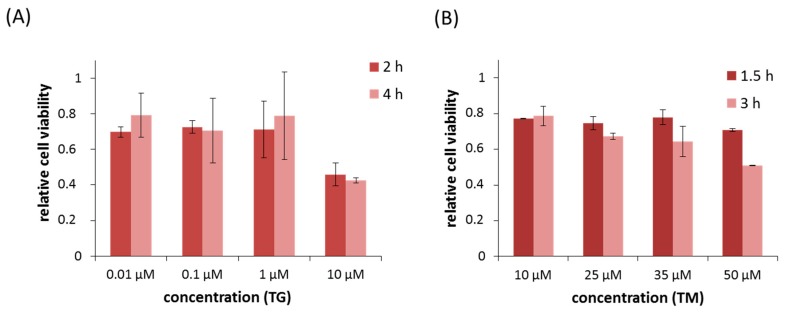
Cell viability decreases with respect to endoplasmic reticulum (ER) stress. Relative viability upon (**A**) thapsigargin (TG); and (**B**) tunicamycin (TM) treatment. HEK293T cells were treated with various concentrations and treatment periods of ER stressors. Cell viability was measured by using cell viability assay. Three parallel experiments were carried out and the average of relative cell viability was plotted (errors bars represent standard deviation).

In order to test the effect of PERK silencing during ER stress, cell viability assays were carried out using the two most well-known ER stressors: thapsigargin (TG) and tunicamycin (TM). TG disrupts the calcium homeostasis, while TM inhibits N-linked glycosylation in the ER [1]. HEK293T is widely used in studies on ER stress and related processes; moreover, the majority of our previous findings were gained on these cells. For the sake of comparability, we chose this cell line also in the present study. We added TG or TM to HEK293T cells using various concentrations and treatment periods to choose a protocol, where cell viability decreased by ≈50%. Treatments of 2 and 4 h were accomplished with 0.01, 0.1, 1, and 10 µM for TG (Figure 1A). We also obtained similar results for 2 and 4 h of treatments, particularly a drastic decrease in cell viability that was observed only at high concentration of ER stressors. TM was added for 1.5 and 3 h to the cell with 10, 25, 35, and 50 µM (Figure 1B). Around ≈50% of viable cells decreased when 35 µM of TM was used for 3 h. According to these viability assays 10 µM TG for 2 h or 35 µM TM for 3 h were added to the cells in our subsequent experiments.

**Figure 2:**
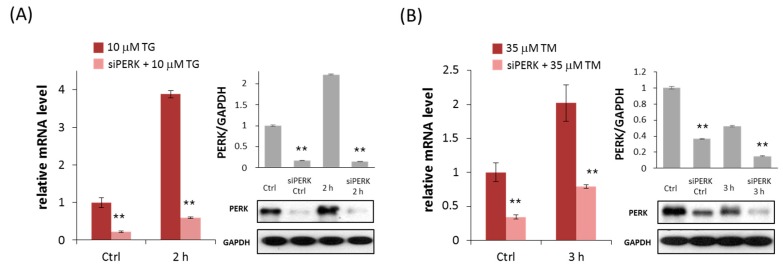

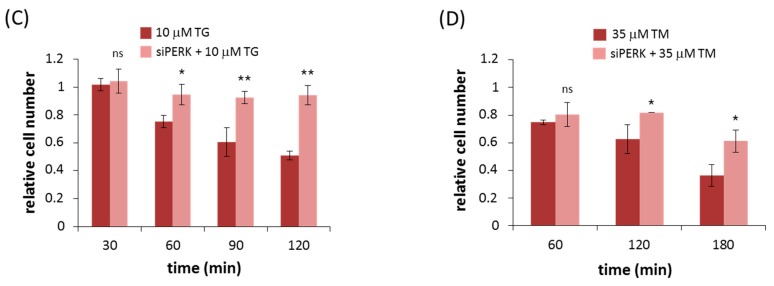
PERK silencing increases cell viability during persistent ER stress. The efficiency of PERK silencing was checked both on mRNA (left panel) and protein (right panel) levels followed in time via (**A**) TG (10 µM) and (**B**) TM (35 µM) treatment. The mRNA level was followed by real-time PCR and the expression level of PERK was followed by Western blot with/without addition of PERK siRNA for 2 (TG) and 3 h (TM) long treatment. GAPDH was used as housekeeping gene. The intensity of PERK is normalized for GAPDH. The amount of viable cells was assessed in PERK-silenced cells after (**C**) TG (10 µM) or (**D**) TM (35 µM) treatment in time. The amount of viable HEK293T cells was followed in time by measuring the percentage of cells permeable to trypan blue. Three parallel experiments were carried out and the amount of viable cells (lower panel) was plotted (errors bars represent standard deviation, asterisks indicate statistically significant difference: * *p* < 0.05; ** *p* < 0.01); ns: not significant.

Before we study the role of PERK downregulation in maintaining cell viability, the effects of PERK silencing on PERK mRNA expression level were tested both in TG- and TM-treated cells (Figure 2A,B). We examined PERK mRNA level by real-time PCR in HEK293T. As expected, transfection with PERK siRNA strongly suppressed the PERK mRNA level during persistent ER stress. While PERK mRNA expression level was gradually increased with respect to ER stressor, the mRNA level in TM- or TG-treated cells transfected with PERK siRNA was ≈25% of the corresponding control. The protein level of PERK was also followed in time with respect to ER stress (Figure 2A,B). These data confirm that PERK silencing effectively suppressed PERK mRNA level in each treatment.

We next tested how PERK silencing influences cell viability during ER stress by counting the amount of viable cells. We transfected HEK293T cells with PERK siRNA, then 10 µM TG (Figure 2C) or 35 µM TM (Figure 2D) was added and the relative amount of viable cells was checked in every 30 min for up to 2 h (TG) or in every 60 min for up 3 h (TM). The amount of viable cells was distinguishable higher from that of untransfected cells after addition of ER stressor. At the end of treatment only 40% of cells were viable of untransfected cells with persistent ER stress, meanwhile more than 60% of cells remained alive when PERK siRNA transfection preceded addition of ER stressor.

These results demonstrated that silencing of PERK significantly extended cell viability with respect to ER stress. Thus, PERK signaling pathway directly affects cell viability in response to ER stress.

### 2.2. Silencing of PERK Extends Autophagy-Dependent Survival and Delays Apoptotic Cell Death during ER Stress

**Figure 3:**
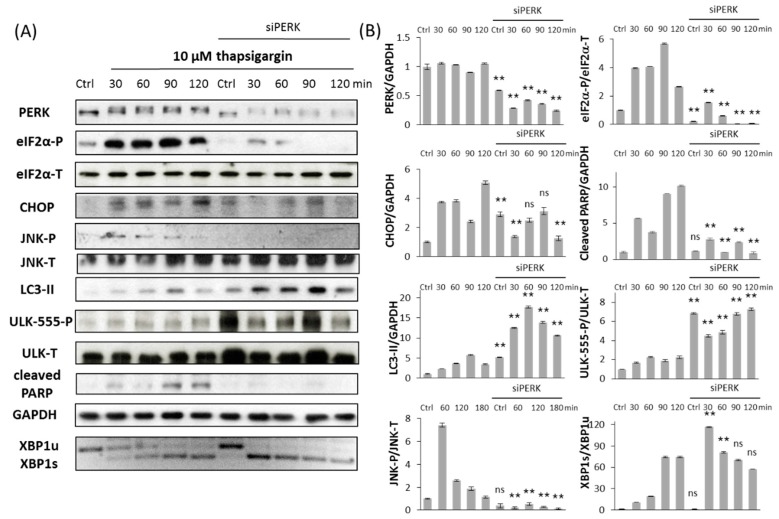
Silencing PERK delays apoptotic cell death at TG treatment. (**A**) Immunoblot results of key regulatory components. HEK293T cells were treated with 10 µM TG for 2 h without/with using PERK siRNA. The expression of the crucial autophagy (LC3II, ULK-555-P), apoptosis (cleaved PARP), PERK (PERK-T, eiF2α-P, CHOP) and IRE-1 (JNK-P, XBP1) markers followed in 30 min intervals by immunoblotting. All the markers except XBP1 were done by Western blot, while splicing of XBP1 was followed by RT-PCR. GAPDH was used as the housekeeping gene. (**B**) Densitometry data of immunoblotting. The intensity of PERK, LC3II, cleaved PARP, CHOP is normalized for GAPDH, eiF2α-P is normalized for total level of eiF2α, ULK-555-P is normalized for total level of ULK-T, JNK-P is normalized for total level of JNK and spliced XBP1 level is normalized for total level of unspliced XBP1. Three parallel experiments were carried out (error bars represent standard deviation, asterisks indicate statistically significant difference: ** *p* < 0.01); ns: not significant.

**Figure 4:**
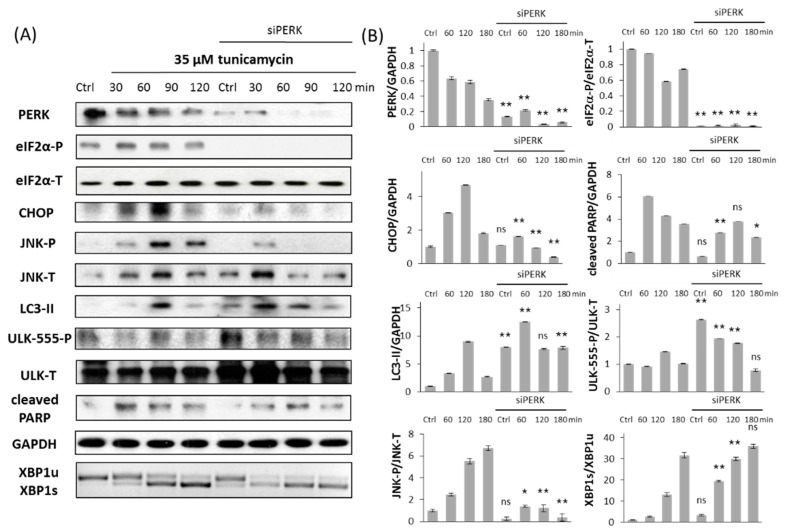
PERK silencing delays apoptotic cell death at TM treatment. (**A**) Immunoblot results of key markers. HEK293T cells were treated with 35 µM TM for 3 h without/with use of siPERK. The expression of the crucial autophagy (LC3II, ULK-555-P), apoptosis (cleaved PARP), PERK (PERK, eiF2α-P, CHOP) and IRE-1 (JNK-P, XBP1) markers were followed every 60 min by immunoblotting. All the markers except XBP1 were done by Western blot, while splicing of XBP1 was followed by RT-PCR. GAPDH was used as housekeeping gene. (**B**) Densitometry data of immunoblotting. The intensity of PERK, LC3II, cleaved PARP, CHOP is normalized for GAPDH, eiF2α-P is normalized for total level of eiF2α, ULK-555-P is normalized for total level of ULK-T, JNK-P is normalized for total level of JNK and spliced XBP1 level is normalized for total level of unspliced XBP1. Three parallel experiments were carried out (errors bars represent standard deviation, asterisks indicate statistically significant difference: * *p* < 0.05; ** *p* < 0.01); ns: not significant.

It is well-known that PERK pathway has an important role in ER stress response mechanism by controlling general protein synthesis. However it remains still unclear whether it promotes autophagy-dependent survival or apoptotic cell death upon prolonged ER stress. Therefore the typical markers of autophagy (LC3-II, ULK-555-P) and apoptosis (cleaved PARP) were studied by immunoblotting when siPERK transfection was followed by TG (Figure 3) or TM (Figure 4) treatment. The effect of ER stressors was monitored in time both in untransfected and siPERK transfected HEK293T cells; samples were collected every 30 min for up to 2 h (TG) or in every 60 min for up to 3 h (TM). To confirm that PERK silencing was successful PERK level was detected (Figure 3 and Figure 4). PERK has a mobility shift in response to addition of ER stressor supposing its activation self-phosphorylation during TG treatment, meanwhile PERK level was significantly reduced after transfection with siPERK. To further confirm the silencing of PERK the downstream targets of PERK arm, including eiF2α-P and CHOP were also presented (Figure 3 and Figure 4).

Untransfected cells have shown a transient increase of LC3-II and ULK-555-P after 30 min of TG or 60 min of TM treatment referring to a transient activation of autophagy with persistent ER stress. This decay of survival mechanism was always followed by apoptotic cell death (see the PARP cleavage within 90 min of TG and 60 min of TM treatment on Figure 3 and Figure 4). By contrast, PERK silencing resulted in prolonged autophagic mechanism. This was detected by high level of both LC3-II and phosphorylated ULK-555 throughout the treatment with ER stressor; meanwhile PARP cleavage was delayed. Since PARP cleavage is promoted by the effector Caspase-3, these data confirm that apoptotic cell death was delayed after transfection with siPERK followed by ER stress. We observed similar results both at TG and TM treatment, suggesting that this phenotype might be general upon prolonged ER stress.

These data demonstrated that HEK293T cells transfecting siPERK significantly improved their survival due to extended autophagy-dependent survival and delayed apoptotic cell death in response to persistent ER stress.

### 2.3. PERK Affects the Apoptosis Inducer of Inositol Requiring Kinase 1 (IRE-1) Arm Positively with Respect to ER Stress

Next we investigated the regulatory connection between PERK and IRE-1 arms of UPR in the control of life-and-death decision during ER stress. To do, this the important targets of IRE-1 (i.e., XBP1s, JNK-P) were also detected in time in HEK293T cells transfected with siPERK. A detailed time course of TG (10 µM) or TM (35 µM) addition was performed for up to 2 h for TG (Figure 3) and 3 h for TM (Figure 4) treatment. The splicing of XBP1 was detected by RT-PCR, while the phosphorylation state of JNK was monitored by immunoblotting.

In untransfected HEK293T cells, XBP1 splicing was observed already at 30 min after TG treatment, when LC3-II also appeared (Figure 3). This result confirms the important role of active Xpb1s during autophagy-dependent survival. Meanwhile JNK phosphorylation was shown within 1.5 h, confirming that JNK has an essential role in the suicide mechanism of cell with persistent ER stress (Figure 3). Interestingly, the dynamical profile of XBP1 splicing is comparable in PERK siRNA transfected cells; however, we detected only a slight transient activation of phosphorylated JNK. Similarly, XBP1 splicing occurs parallel with autophagy activation in TM-treated cells transfected with or without siPERK (Figure 4). Although addition of TM results in a drastic phosphorylation of JNK within 2 h, silencing of PERK largely suppresses JNK-P with persistent ER stress (Figure 4).

Although XBP1 splicing is not affected, the IRE-1 target apoptosis inducer, JNK-P has only a transient activation when PERK pathway is not working during ER stress. Only a modest transient peak of JNK phosphorylation is observed suggesting that the PERK arm of UPR has an essential role in promoting the IRE-1-dependent suicide loop of life-and-death decision upon excessive level of ER stress.

### 2.4. The Switch-Like Activation of Apoptosis Inducers Is Precisely Regulated during ER Stress

**Figure 5:**
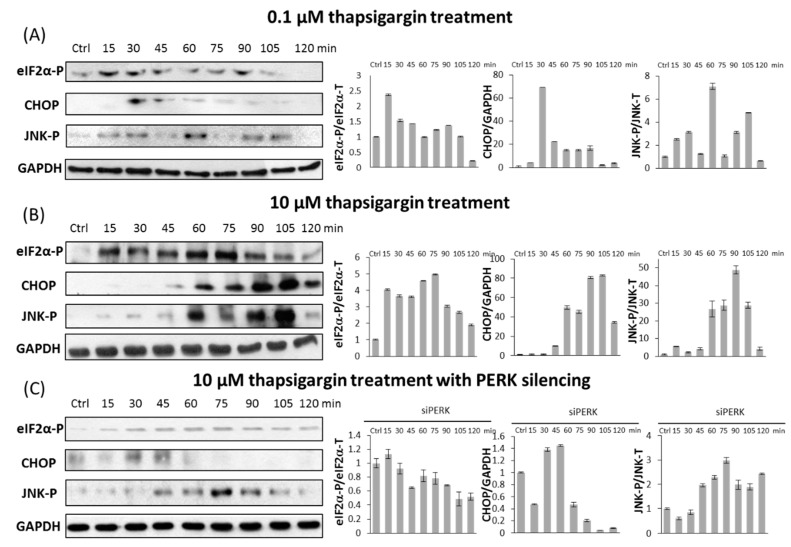
Kinetics of apoptosis inducers with respect to ER stress. HEK293T cells were treated with (**A**) low level of TG (0.1 µM); (**B**) high level of TG (10 µM); and (**C**) high level of TG (10 µM) combined with PERK silencing. The expression level of eiF2α-P, CHOP and JNK-P markers were followed in every 15 min for 2 h by immunoblotting. *GAPDH* was used as a housekeeping gene. Immunoblot results (left panel) and their densitometry data (right panel) are depicted. The intensity of CHOP is normalized for GAPDH, eiF2α-P is normalized for total level of eiF2α and JNK-P is normalized for total level of JNK. The total level of eiF2α and JNK-P are not shown. Three parallel experiments were carried out (errors bars represent standard deviation).

To further examine the activation kinetics of both PERK and IRE-1-controlled apoptosis inducers (i.e., CHOP, JNK-P), with respect to ER stress, we performed a detailed time course by treating HEK293T cells with 0.1 or 10 µM of TG for up to 2 h (Figure 5A,B). Samples were taken every 15 min, and the whole-cell lysates were assessed for immunoblotting. To confirm that the PERK branch of the UPR response mechanism was active during treatment, eiF2α phosphorylation was detected in time. According to previously published data, both low and high levels of ER stress resulted in a transient phosphorylation of eiF2α upon prolonged ER stress.

Low level of ER stress does not affect cell viability (Figure 1A) suggesting that apoptotic cell death cannot be turned on. However, we observed a modest transient activation of both PERK and IRE-1-controlled apoptosis inducers with persistent ER stress (Figure 5A). CHOP became active after 30 min, while JNK-P was observed after 60 min, although both of them quickly disappeared. Their activation was much more intense and assumed to have a switch-like activation profile, when we treated HEK293T cells with 10 µM of TG (Figure 5B). These results suggest that apoptosis inducers of both PERK and IRE-1 arms of UPR get activated even at a low level of ER stress, but their activity is not strong enough to accelerate the suicide mechanism. The concentration of ER stressor has to reach a critical threshold to result in a switch-like activation of apoptosis inducers (see CHOP and JNK-P levels in Figure 5B) followed by apoptotic cell death. Similar effects were observed by TM treatment (data not shown).

Interestingly, transfection with PERK siRNA combined with TG treatment (10 µM for up to 2 h) results in a transient activation of apoptosis inducers similar to permanent treatment with a low level of ER stressor (Figure 5C). Due to the PERK silencing, we observed only a small amount of CHOP activated after 30 min treatment and it quickly disappeared. JNK was phosphorylated after 75 min, but it could not remain active either. Both apoptosis inducers became inactivated within 15 min after their induction suggesting that apoptotic cell death did not turn on with persistent high level of ER stress. The switch-like characteristic of induction of apoptosis inducers diminished; only a reduced transient activation peak was observed in the absence of PERK. These results confirm that apoptotic cell death is delayed in cells transfected with PERK siRNA due to the imperfect activation of apoptosis inducers even at high levels of ER stress. Our results suggest that this dynamical activation profile of PERK and IRE-1-controlled apoptosis inducers (i.e., CHOP and JNK-P) is indistinguishable to that seen in cells treated with low level of ER stress.

### 2.5. A Mathematical Model Suggests a Positive Feedback Loop between IRE-1 (Inositol Requiring Protein 1) and PERK (Protein Kinase (RNA)-Like Endoplasmic Reticulum Kinase)

**Figure 6:**
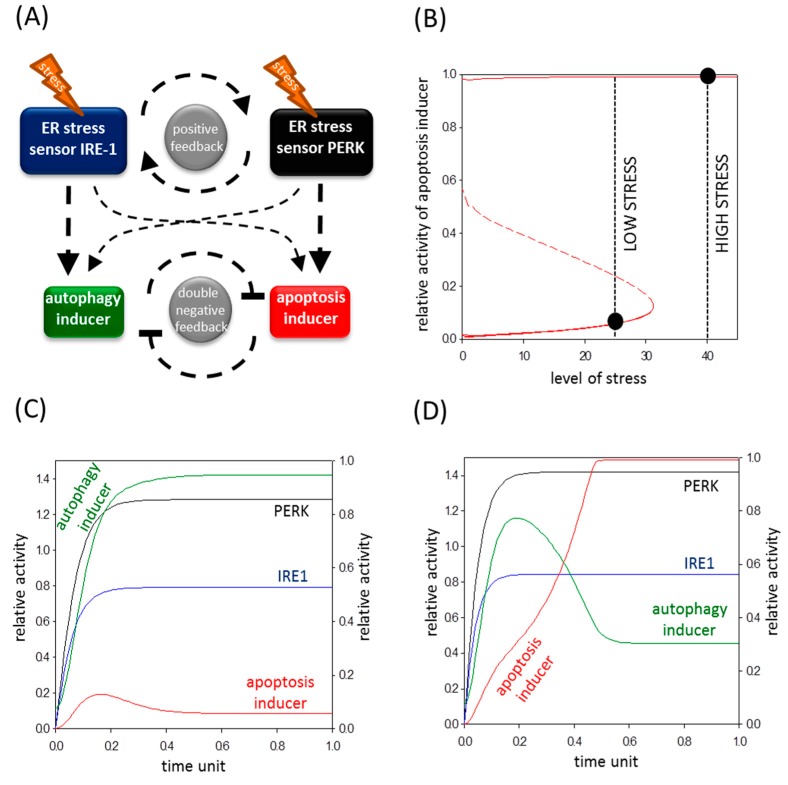
Feedback loops guarantee the irreversibility of life-and-death decision. (**A**) The wiring diagram of control network. The autophagy inducer, the apoptosis inducer, the ER stress sensor PERK and the ER stress sensor IRE-1 are denoted by isolated green, red, black and blue boxes, respectively. Dashed line shows how the molecules can influence each other; (**B**) Signal response curve of life-and-death decision. The signal-response curve of apoptosis inducer is shown with respect to the increasing stress level. Solid lines denote stable states, while dashed line denotes the unstable state. Black dots represent the steady states at both low and high level of ER stress. The computer simulation of (**C**) low (stress = 25) and (**D**) high (stress = 40) level of ER stress. The temporal dynamics is simulated for time unit after ER stress was generated. The dynamics of ER stress sensor PERK, ER stress sensor IRE-1, autophagy and apoptosis inducers are plotted.

To investigate further the role of IRE-1 and PERK in determining the kinetical feature of life-and-death decision during persistent ER stress, a theoretical analysis was carried out. Recently, a mathematical model was published by our lab [12]. That model precisely explains how cells choose between autophagy-dependent survival and apoptotic cell death where their inducers are crosslinked with a double-negative feedback loop. Similar to that model, we assumed here a double-negative feedback loop between autophagy and apoptosis controlled by autophagy and apoptosis inducers, respectively (see the wiring diagram in Figure 6A). However, we rewired the simple model according to the new experimental data. In this case, two ER stress-induced ER stress sensors are distinguished, representing the PERK and IRE-1 arms of UPR, respectively. It is well known that both pathways act positively on autophagy and apoptosis inducers, too. Since they have already experimentally proven that inhibition of the IRE-1 arm decreases cell viability, we suppose that IRE-1 is much stronger on autophagy than on apoptosis inducers (see the thin and thick arrows rising from ER stress sensor IRE-1 in Figure 6A). Our data confirm that silencing of PERK increases cell viability; therefore, we assume that PERK-dependent activation is more intense on an apoptotic inducer compared to an autophagy inducer (see the thin and thick arrows rising from ER stress sensor PERK on Figure 6A). Deegan et al. [20] proved that IRE-1 enhances the PERK pathway, whereas our results have shown that PERK also has a positive effect on IRE-1 generating a positive feedback loop between the two ER stress sensors in the regulatory system.

Our wiring diagram used for computer simulation of the experiments is shown in Figure 6A. To get the dynamical characteristic of life-and-death decision with respect to ER stress first a signal-response curve was generated (Figure 6B). The experiments suggest that human cells can occupy either the state of autophagy-dependent survival or apoptotic cell death after ER stress. This indicates that the apoptosis inducer has two alternative steady states in the function of increasing ER stress, namely, it has a bistable characteristic (see red lines in Figure 6B). Bistability is an emergent property of the stress response mechanism generated by the non-linear characteristic of double-negative feedback loop between autophagy and apoptosis inducers. The two stable steady states of apoptosis inducer (i.e., inactive and active) are separated by an unstable regime, where the system cannot settle for a long time (see red dashed lines in Figure 6B). Although the unstable state cannot be observed normally, it has an essential role in determining the experimental results. At low level of ER stress, the ER stress response sensors (i.e., PERK and IRE-1) activate the autophagy inducer, whereas the cell death mechanism remains inactive (Figure 6B,C). Although apoptosis inducers (such as CHOP and JNK-P) try to be active, they are quickly downregulated by the survival mechanism (see Figure 5A and Figure 6C) and the system occupies its lower steady state (see black dot at low stress in Figure 6B). However, when the level of ER stress reaches a threshold value, the switch-like activation of apoptosis inducers can win against autophagy (see Figure 5B and Figure 6B,D) and the system jumps into its higher steady state (see black dot at high stress in Figure 6B). Although the survival mechanism always has a short window when it is active, excessive level of ER stress is able to switch on the suicide loop. Our model confirms that the switch-like characteristic of the apoptosis inducer is determined by a double-negative feedback loop between autophagy-dependent survival and apoptotic cell death.

### 2.6. The Altered Upregulation of Autophagy and Apoptosis Inducers by IRE-1 and PERK Determines the Life-and-Death Decision

**Figure 7:**
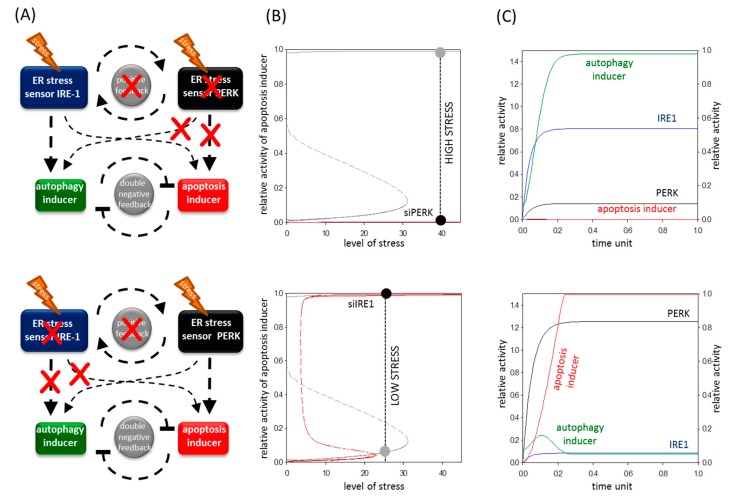
The absence of positive feedback loop between ER stress sensors affects the life-and-death decision. (**A**) The wiring diagram of control network when ER stress sensor PERK (upper panel) is silenced or ER stress sensor IRE-1 (lower panel) is inhibited. The autophagy inducer, the apoptosis inducer, the ER stress sensor PERK and the ER stress sensor IRE-1 are denoted by isolated green, red, black and blue boxes, respectively. Dashed line shows how the molecules can influence each other. Red cross depicts the missing regulatory connections in the absence of PERK or IRE-1; (**B**) Signal response curve of life-and-death decision. The signal-response curve of apoptosis inducer is shown with respect to the increasing stress level in siPERK-transfected (red line on upper panel) or IRE-1-(red line on lower panel) inhibited cells. The grey curve depicts the original signal response curve. Solid lines denote stable states, while dashed line denotes the unstable state. Black dot represents the steady state at high level of ER stress in the absence of PERK or IRE-1; (**C**) The computer simulation of siPERK (upper panel PERKT = 0.2) or IRE-1 inhibition (lower panel IRE1T = 0.1) at high (stress = 40) level of ER stress. The temporal dynamics is simulated for time unit after ER stress was generated. The dynamics of ER stress sensor PERK, ER stress sensor IRE-1, autophagy and apoptosis inducers are plotted.

In order to qualitatively explore the contribution of positive feedback loop between IRE-1 and PERK in the ER stress response mechanism of cells, both ER stress sensors were diminished separately. First we reduced the total amount of PERK to 10% mimicking the transfection of cells with siPERK. Our experimental result suggests that silencing of PERK delays apoptotic cell death with respect to ER stress (Figure 3 and Figure 4). The absence of PERK diminishes its strong positive effect on apoptosis inducer and a weaker positive effect on autophagy inducer is also missing (Figure 7A, upper panel). In this case, the activation threshold of apoptosis inducer moves to the right on the signal response curve (Figure 7B, upper panel).

Therefore, the ER stress has to reach a much higher level to switch on the suicide loop and subsequently generating a delayed cell death. The computer simulation shows that active IRE-1 alone can enhance the autophagy inducer resulting in an efficient survival mechanism; however, the apoptosis does not turn on (Figure 7C, upper panel).

When IRE-1 level was reduced to 10% an interesting dynamical feature was observed on the signal response curve (Figure 7A,B, lower panel). Since IRE-1 is much stronger on an autophagy inducer than on an apoptosis inducer, the activation threshold of the apoptosis inducer moves to the left on the curve. This means that a lower ER stress level might be enough to switch on the apoptotic cell death when only the PERK branch is active. Our model confirms the well-known experimental data that inhibition of IRE-1 results in early entry suicide mechanism. When IRE-1 is blocked, the window of autophagy-dependent survival is shorter; after a short, transient period of autophagy, apoptosis inducers are quickly activated with persistent ER stress (Figure 7C, lower panel).

Inactivation of either PERK or IRE-1 arms of UPR annihilates the positive feedback loop between them. Our model demonstrates that the absence of this positive feedback loop has a very dangerous consequence. Namely, the threshold required for turning off the apoptosis inducer is moved to the positive regime of signal response curve (see Figure 7B). Therefore, the suicide loop is not irreversible, and the one-directionality of one of the most serious cell mechanisms is no longer guaranteed with respect to ER stress. Our model confirms that the PERK-dependent IRE-1 regulation proved experimentally in this study has a crucial role in determining the dynamical features of the control network during an excessive level of ER stress.

## 3. Discussion

Choosing between life and death is one of the most important tasks of cells building up an organism. It is well-known that accumulation of misfolded proteins in the endoplasmic reticulum due to various ER stress events leads to the activation of unfolded protein response (UPR). The primer role of the three arms of UPR is to reduce the bulk of damages and try to drive back the system to the former or a new homeostatic state by autophagy-controlled self-eating, while excessive stress results in apoptotic cell death [4]. In this study we focused on the kinetical features of PERK branch of UPR and the contribution of IRE-1 and PERK arms in life-and-death decision. Here both theoretical and molecular biological techniques were incorporated to explore the dynamical behaviours of the control network upon prolonged ER stress.

It has already been proven that autophagy-dependent survival is followed by apoptotic cell death during a high level of ER stress [8]; here, we further confirmed that the two processes could not be active at the same time (Figure 3 and Figure 4). Therefore, corresponding with our previous data [12], a double-negative feedback loop was assumed between the autophagy and apoptosis inducers of stress response system guarantying a mutual antagonism between the two mechanisms (Figure 6A). This double-negative feedback loop ensures that autophagy inducers have a transient activation followed by the switch-like activation of apoptosis inducers (see CHOP and JNK-P levels in Figure 5B) when ER stress level reaches a critical value (Figure 6).

Similar to already published data [33,34], we observed enhanced cell survival when ER stress was combined with PERK silencing (Figure 2). The experimental data performed here confirms that autophagy remains active during excessive levels of ER stress, while the apoptotic cell death is delayed in PERK siRNA-transfected cells (Figure 3 and Figure 4). Thus, the “window of autophagy-dependent survival” is extended in the absence of a PERK branch of UPR. Although the PERK-induced apoptosis inducers (i.e., CHOP) had a switch-like activation profile at an excessive level of ER stress, it had only an insufficient transient activation peak in PERK siRNA-transfected cells followed by the addition of an ER stressor, thereby demonstrating that the stress response mechanism could not turn on apoptosis when the PERK branch was missing (Figure 3, Figure 4 and Figure 5C). The transient activation of apoptosis inducers suggests that PERK is essential for a suicide mechanism during persistent ER stress. Similar behaviour was observed when cells were treated with a tolerable level of ER stress, i.e. autophagy remained effective, while the apoptosis inducers had only a transient activation peak, though cell death was not observed (see CHOP and JNK-P levels in Figure 5A). Our experimental data claim that two criteria must be fulfilled to switch from autophagy-dependent survival to apoptotic cell death upon prolonged ER stress: (1) the stress level has to reach a critical threshold; (2) PERK branch of UPR has to be active.

To explore the relative contributions of both PERK and IRE-1 controlling life-and-death decision during ER stress, two ER stress sensors were built into our model, representing the PERK and IRE-1 arms of UPR, respectively. It is well known that both PERK and IRE-1 branches are able to enhance autophagy and apoptosis inducers, but we claim that their intensity on their targets must be different. Since inhibition of IRE-1 drastically decreases cell viability by inducing apoptotic cell death [35], a more intensive positive effect is assumed on its autophagy inducer than on apoptosis inducer in our model (see dashed arrows in Figure 6A). However here we confirmed that silencing of PERK combined with ER stress resulted in delayed apoptotic cell death. Therefore PERK seems to be more effective on its apoptosis inducers than on autophagy inducers (see dashed arrows in Figure 6A). Deegan et al. have already proven that apoptosis inducers of PERK are weaker when IRE-1 is inhibited [20]. Here we demonstrated that PERK silencing decreases the phosphorylation state of JNK (Figure 3, Figure 4 and Figure 5B), thereby validating a PERK-dependent positive effect on IRE-1 arm with respect to ER stress. Building these connections into our model, a positive feedback loop formed between PERK and IRE-1 arms of UPR (Figure 6A).

Our simple model of a stress response mechanism is able to explain the alteration of life-and-death decision in the absence of either PERK or IRE-1 branches. Since PERK has a significant effect on the apoptosis inducer, silencing of PERK moved the signal response curve of the apoptosis inducer to the right (Figure 7A,B, upper panel). Namely, the activation threshold of the suicide mechanism got to a higher stress value, resulted in a delayed apoptotic cell death (Figure 7B,C, upper panel). Here, we confirmed this effect of PERK with two various ER stressors (Figure 3 and Figure 4). However, IRE-1 inhibition resulted in early apoptotic cell death upon prolonged ER stress [35]. Since IRE-1 has a pronounced positive effect on autophagy inducer, here we show that the double-negative feedback loop between autophagy and apoptosis inducers got attenuated in the absence of IRE-1 (Figure 7A, lower panel). The enervate autophagy inducer cannot maintain the inactive state of the apoptosis inducer; therefore, the survival mechanism quickly turns off and the activation threshold of suicide mechanism moves to a lower stress value (Figure 7B,C, lower panel). Our results suggest that the presence of both IRE-1 and PERK branches of UPR are essential to guarantee a sufficient window for autophagy-dependent survival followed by apoptotic cell death upon prolonged ER stress.

Our theoretical analysis assumes that the absence of either IRE-1 or PERK has another important effect on life-and-death decision during ER stress. The activation threshold of the apoptosis inducer also moves to the right on the signal response curve when one of the branches of UPR is missing (Figure 7B). Namely, the irreversible apoptosis induction becomes reversible during persistent ER stress. Irreversible switch guarantees that once a cell is engaged in apoptosis induction, it never returns to its previous autophagy-dependent survival state, even if the cellular stressor is washed out from the cell. This irreversibility of the control network is essential to avoid the proliferation of severely damaged cells [13]. Our model suggests that downregulation of either IRE-1 or PERK results in a reversible induction of apoptosis inducer with respect to ER stress, which generates a significant alteration of the regulatory system. The control network can no longer guarantee the single direction of the suicide mechanism. Thus, we suggest that the positive feedback loop between the arms of UPR is crucial to produce a point of no return for the decision-making process between life and death. In the near future, to confirm the results of our theoretical analysis, we will study this reversible characteristic of apoptosis induction in the absence of one arm of UPR by washing out the excessive level of ER stressor.

Since ER stress is involved in various human pathologies such as neurodegenerative diseases, obesity, diabetes and many others, studying ER stress-induced life-and-death decision has medical importance. Our result shows that proper silencing of one of the arms of UPR can alter the control network of life-and-death decision, i.e. it can either extend autophagy-dependent survival or speed up the cell death mechanism. According to the ER stress-dependent disease, this knowledge might be used later to elaborate a precise medical treatment for the patient.

## 4. Materials and Methods

### 4.1. Materials

Thapsigargin (Sigma-Aldrich, St. Louis, MO, USA, T9033) and tunicamycin (Sigma-Aldrich, T7765) were purchased. All other chemicals were of reagent grade.

### 4.2. Cell Culture and Maintenance

As a model system, human embryonic kidney (HEK293T, ATCC, CRL-3216) cell lines were used, maintained in DMEM (Life Technologies, Carlsbad, CA, USA, 41965039) medium supplemented with 10% fetal bovine serum (Life Technologies, 10500064) and 1% antibiotics/antimycotics (Life Technologies, 15240062). Culture dishes and cell treatment plates were kept in a humidified incubator at 37 °C in 95% air and 5% CO_2_.

### 4.3. Sodium Dodecyl Sulfate Polyacrylamide Gel Electrophoresis (SDS-PAGE) and Western Blot Analysis

Cells were harvested and lysed with 20 mM Tris, 135 mM NaCl, 10% glycerol, 1% NP40, pH 6.8. Protein content of cell lysates was measured using Pierce BCA Protein Assay (Thermo Scientific, Waltham, MA, USA, 23225). During each procedure equal amounts of protein were used. SDS-PAGE was done by using Hoefer miniVE (Amersham, UK). Proteins were transferred onto Millipore (Billerica, MA, USA) 0.45 µm PVDF membrane. Immunoblotting was performed using TBS Tween (0.1%), containing 5% non-fat dry milk for blocking membrane and for antibody solutions. Loading was controlled by developing membranes for GAPDH or dyed with Ponceau S in each experiment. The following antibodies were applied: antiLC3B (SantaCruz, Santa Cruz, CA, USA, sc-16755), antiPARP (Cell Signaling, Danvers, MA, USA 9542S), antiGADD 153 (SantaCruz, sc-7351), antiCREB-2 (SantaCruz, sc-200), antiP-c-Jun (Cell Signaling, 9261S), antic-Jun (Cell Signaling, 9165S), antiPERK (Cell Signaling, 3192S), antiULK1 (Cell Signaling, 8054S), antiP-ULK1 (S555) (Cell Signaling, 5869S), antieIF2α (Cell Signaling, 9722S9), antiP-eIF2α (Cell Signaling, 9721L), and antiGAPDH (Santa Cruz, 6C5), HRP conjugated secondary antibodies (SantaCruz, sc-2020 and Cell Signaling, 7074S, 7076S).

### 4.4. Statistics

For densitometry analysis, Western blot data were acquired using ImageQuant 5.2 software. The relative band densities were shown and normalized to an appropriate GAPDH band used as reference protein (see Appendix A). Results are presented as mean values ± S.D. and were compared using ANOVA with Tukey’s multiple comparison post hoc test. Asterisks indicate statistically significant difference from the appropriate control: * *p* < 0.05; ** *p* < 0.01.

### 4.5. RNA Interference

RNA interference experiments were performed using Lipofectamine RNAi Max (Invitrogen, Carlsbad, CA, USA) in GIBCO™ Opti-MEM I (GlutaMAX™-I) Reduced-Serum Medium liquid (Invitrogen) and 20 pmol/mL siRNA. The siPERK oligonucleotides were purchased from Eurofins Genomics (the oligonucleotide sequence: 5′-GUGACGAAAUGGAACAAGA(dTdT)-3′). A total of 200,000 HEK293T cells were incubated at 37 °C in a CO_2_ incubator in antibiotic-free medium for 42 h, then the RNAi duplex-Lipofectamine™ RNAiMAX complexes were added to the cells for 24 h. Then fresh medium was added to the cells and the appropriate treatment was carried out.

### 4.6. Reverse Transcription-Polymerase Chain Reaction (RT-PCR)

Total RNA content of cells was extracted using TRIzol RNA isolation reagent (Invitrogen) [41]. To amplify the spliced and unspliced XBP1, and the *GAPDH* genes, Thermo Scientific™ (2X) PCR Master Mix (contains 0.05 U/µL Taq DNA polymerase, reaction buffer, 4 mM MgCl_2_, 0.4 mM of each dNTP) and primers were used. The primers are as follows: for XBP1: (forward) 5′-CCTTGTAGTTGAGAACCAGG-3′ and (reverse) 5′-GGGCTTGGTATATATGTGG-3’, for GAPDH: (forward) 5′-TGCACCACCAACTGCTTAGC-3′ and (reverse) 5′-GGCATGGACTGTGGTCATGAG-3′. The PCR thermocycles were the followings: 95 °C 10 min (1×), (95 °C 30 s, annealing temperature 45 s, 72 °C 30 s) (40×), 95 °C 5 min, 55 °C 1 min, 97 °C 30 s (1×). The annealing temperature of XBP1 and GAPDH primers were 57 and 58 °C. The XBP1 and GAPDH PCR products were electrophoresed on 3% or 1% agarose gel. GAPDH was used as a loading control.

### 4.7. Real-Time PCR

Total RNA content of cells was extracted using TRIzol RNA isolation reagent (Invitrogen) [41]. Retrotranscription was performed using SuperScriptII First-Strand Synthesis System (Invitrogen). Nucleic acid levels were measured using NanoDrop2000 UV calculator. Equal amounts of cDNA were used for real-time PCR to check the efficiency of PERK silencing. PCR reaction and real-time detection was performed using GoTaq(R) qPCR Master Mix (Promega, Madison, WI, USA, A6002) and STRATAGENE Mx3005P Real-Time PCR Detection System. The real-time PCR thermocycles were the followings: 95 °C 10 min (1×), 95 °C 30 s, 58 °C 45 s, 72 °C 30 s (40×), 95 °C 5 min, 55 °C 1 min, 97 °C 30 s (1×). The appropriate forward and reverse real-time PCR primers were used for PERK and GAPDH.

### 4.8. Cell Viability Assays

The relative amount of viable cells was calculated by Burker chambers. Cell viability was detected using CellTiter-Blue assay (Promega, G8080). Cells were grown and treated on 96-well plates, and were incubated with resazurin for 2 h at 37 °C. Absorbance was measured at 620 nm, and expressed in arbitrary unit, being proportional to cell toxicity. For each of these experiments at least three parallel measurements were carried out.

### 4.9. Mathematical Modeling

The regulatory network was translated into a set of nonlinear ordinary differential equations (ODEs) and analysed using the techniques of dynamical system theory [42,43,44]. The parameter values are chosen in such way to capture all the possible qualitative behaviours that the given network can exhibit. Dynamical simulations were carried out using the program XPPAUT, which is freely available from http://www.math.pitt.edu/~bard/xpp/xpp.html [43,44]. We provide the XPP codes that can be used to generate all the figures in the manuscript (see Appendix A).

## Appendix A. The Minimal Model of ER Stress Response Mechanism

A biological network can be translated into a set of ordinary differential equation (ODE) that describes how each component concentration/activity in the network changes with the time. A generic differential equation depicting the temporal changes of a regulatory component is composed of two parts: production and consumption terms. The production can be given by protein synthesis and/or an activation term, while the consumption can be given by protein degradation and/or inactivation term. Usually synthesis, degradation, binding and dissociation reactions are described by mass action kinetics, whereas protein activity can be described either by mass action or Michaelis-Menten kinetics. Solving a set of non-linear ODEs gives the time evolution of the protein concentration/activity (time courses) or the input-output relationship can be also obtained (signal response curves).

The temporal profiles and signal response curves were computed numerically using *XPP-AUT*. All the simulations presented in the text are based on the following XPP codes which contains ODEs. The rate constants (*k*) have the dimension of min^−1^ and Michaelis constants (*J*) are dimensionless. The proteins levels/activities are given in arbitrary units (a.u).

### Appendix A.1. The Code for Simulating Time Series

# a model to simulate ER stress dependent life and death decision with XPP-Aut

# initial conditions to simulate ER stress dependent life and death decision

init PERK = 0, IRE1 = 0, Apoa = 0, Auta = 0.075

# differential equations

# PERK depicts the PERK branch of UPR

PERK’ = (kaperk’*STRESS + kaperk”*IRE1)*(PERKT-PERK) − kiperk*PERK

# IRE1 depicts the IRE1 branch of UPR

IRE1’ = (kaire1’*STRESS + kaire1”*PERK)*(IRE1T-IRE1) − kiire1*IRE1

# Apoa represents apoptosis inducers (i.e., CHOP, JNK-P) of stress response mechanism

Apoa’ = (kaap + kaap’*PERK + kaap”*IRE1)*(Apot-Apoa)/(Jap + Apot-Apoa) − (kiap + kiap’*Auta)*Apoa/(Jap + Apoa)

# Auta represents autophagy inducers of stress response mechanism

Auta’ = (kaau + kaau’*PERK + kaau”*IRE1)*(Autt-Auta)/(Jau + Autt-Auta) − (kiau + kiau’*Apoa)*Auta/(Jau + Auta)

# parameters

# simulating low level of ER stress: stress = 25

# simulating high level of ER stress: stress = 40

# simulating PERK silencing: PERKT = 0.2, simulating IRE1 inhibition: IRE1T = 0.1

p stress = 0

p kaperk’ = 0.3, kaperk” = 7.5, kiperk = 7.5, PERKT = 2

p kaire1’ = 0.5, kaire1” = 5, kiire1 = 5, IRE1T = 1

p Apot = 1, Jap = 0.01, kaap = 0, kaap’ = 7.5, kaap” = 0.3, kaap”’ = 0, kiap = 2, kiap’ = 10

p Autt = 1, Jau = 0.5, kaau = 0.2, kaau’ = 2, kaau” = 20, kiau = 1, kiau’ = 30

#numerics

@ TOTAL = 1, METH = stiff, XLO = 0, XHI = 1, YLO = 0, YHI = 1, BOUND = 100

done

### Appendix A.2. The Code for Simulating Signal Response Curves

# a model to generate signal response curve of ER stress dependent life and death decision with XPP-Aut

# initial conditions to simulate ER stress dependent life and death decision

init Apoa = 0, STRESS = 0

# differential equations

# Apoa represents apoptosis inducers (i.e., CHOP, JNK-P) of stress response mechanism

Apoa’ = (kaap + kaap’*PERK + kaap”*IRE1)*(Apot-Apoa)/(Jap + Apot-Apoa) − (kiap + kiap’*Auta)*Apoa/(Jap + Apoa)

# STRESS represents the intensity of ER stress

STRESS’ = 0

# steady state functions

# PERK depicts the PERK branch of UPR

PERK = (kaperk’*STRESS + kaperk”*IRE1)*PERKT/(kaperk’*STRESS + kaperk”*IRE1 + kiperk)

# IRE1 depicts the IRE1 branch of UPR

IRE1 = (kaire1’*STRESS + kaire1”*PERK)*IRE1T/(kaire1’*STRESS + kaire1”*PERK + kiire1)

# Auta represents autophagy inducers of stress response mechanism

Auta = Autt*GK(kaau + kaau’*PERK + kaau”*IRE1,kiau + kiau’*Apoa,Jau,Jau)

# ‘Goldbeter-Koshand’ function (GK)

GB(arg1,arg2,arg3,arg4) = arg2-arg1+arg2*arg3+arg1*arg4

GK(arg1,arg2,arg3,arg4) = 2*arg1*arg4/(GB(arg1,arg2,arg3,arg4) + sqrt(GB(arg1,arg2,arg3,arg4)^2-4*(arg2-arg1)*arg1*arg4))

# parameters

# simulating low level of ER stress: stress = 25

# simulating high level of ER stress: stress = 40

# simulating PERK silencing: PERKT = 0.2, simulating IRE1 inhibition: IRE1T = 0.1

p stress = 0

p kaperk’ = 0.3, kaperk" = 7.5, kiperk = 7.5, PERKT = 2

p kaire1’ = 0.5, kaire1” = 5, kiire1 = 5, IRE1T = 1

p Apot = 1, Jap = 0.01, kaap = 0, kaap’ = 7.5, kaap” = 0.3, kaap”’ = 0, kiap = 2, kiap’ = 10

p Autt = 1, Jau = 0.5, kaau = 0.2, kaau’ = 2, kaau” = 20, kiau = 1, kiau’ = 30

done
